# Real-World Implementation of Neurosurgical Enhanced Recovery After Surgery Protocol for Gliomas in Patients Undergoing Elective Craniotomy

**DOI:** 10.3389/fonc.2022.860257

**Published:** 2022-05-24

**Authors:** Yuan Wang, Ya-Fei Xue, Bin-Fang Zhao, Shao-Chun Guo, Pei-Gang Ji, Jing-Hui Liu, Na Wang, Fan Chen, Yu-Long Zhai, Yue Wang, Yan-Rong Xue, Guo-Dong Gao, Yan Qu, Liang Wang

**Affiliations:** ^1^ Department of Neurosurgery, Tangdu Hospital, Airforce Medical University, Xi’an, China; ^2^ Department of Health Statistics, Airforce Medical University, Xi’an, China; ^3^ National Time Service Center, Chinese Academy of Sciences, Xi’an, China; ^4^ School of Optoelectronics, University of Chinese Academy of Sciences, Beijing, China

**Keywords:** gliomas, enhanced recovery after surgery (ERAS), perioperative care, outcomes, craniotomy

## Abstract

**Objective:**

To design a multidisciplinary enhanced recovery after surgery (ERAS) protocol for glioma patients undergoing elective craniotomy and evaluate its clinical efficacy and safety after implementation in a tertiary neurosurgical center in China.

**Methods:**

ERAS protocol for glioma patients was developed and modified based on the best available evidence. Patients undergoing elective craniotomy for treatment of glioma between September 2019 to May 2021 were enrolled in a randomized clinical trial comparing a conventional neurosurgical perioperative care (control group) to an ERAS protocol (ERAS group). The primary outcome was postoperative hospital length of stay (LOS). Secondary outcomes were 30-day readmission rate, postoperative complications, duration of the drainage tube, time to first oral fluid intake, time to ambulation and functional recovery status.

**Results:**

A total of 151 patients were enrolled (ERAS group: n = 80; control group: n = 71). Compared with the control group, postoperative LOS was significantly shorter in the ERAS group (median: 5 days vs. 7 days, p<0.0001). No 30-day readmission or reoperation occurred in either group. The time of first oral intake, urinary catheter removal within 24 h and early ambulation on postoperative day (POD) 1 were earlier and shorter in the ERAS group compared with the control group (p<0.001). No statistical difference was observed between the two groups in terms of surgical- and nonsurgical-related complications. Functional recovery in terms of Karnofsky Performance Status (KPS) scores both at discharge and 30-day follow-up was similar in the two groups. Moreover, no significant difference was found between the two groups in the Hospital Anxiety and Depression Scale (HADS) scores.

**Conclusion:**

The implementation of the ERAS protocol for glioma patients offers significant benefits over conventional neurosurgical perioperative management, as it is associated with enhancing postoperative recovery, without additional perioperative complications and risks.

**Clinical Trial Registration:**

Chinese Clinical Trial Registry (http://www.chictr.org.cn/showproj.aspx?proj=42016), identifier ChiCTR1900025108

## Introduction

Glioma is the most frequent primary malignant brain tumor in adults. Despite significant advances in treatment, the prognosis remains poor. Glioblastoma (GBM) is the most common and lethal subtype, accounting for 49.1% of primary malignant brain tumors. Its median survival time is about 14.4 months (1). Advancements in standard treatment regimens, including maximal safe resection, radiotherapy with concomitant temozolomide (TMZ), maintenance chemotherapy, tumor treatment field (TTF), immunotherapy and targeted therapy, have improved survival rates in patients with low- and high-grade gliomas in the past few decades. Therefore, assessment and improvement of the patient’s functional status in each treatment section have been emphasized.

Enhanced recovery after surgery (ERAS) protocols have been widely adopted in diverse surgical specialties. As a patient-centered perioperative model, ERAS offers several clinical advantages, including reduced surgical stress response, accelerated postoperative recovery, shortened hospital length of stay (LOS) and improved overall satisfaction (2, 3). In addition, our previous study confirmed the feasibility and safety of the ERAS protocol in neurosurgery (4). However, despite the diversity of neurosurgical diseases, there is a paucity of literature on ERAS protocols for various neurological diseases, especially gliomas.

Herein, we designed and implemented a multidisciplinary ERAS protocol for glioma patients undergoing elective craniotomy and evaluated its clinical efficacy and safety in a large tertiary hospital in China. The effect of ERAS protocol on perioperative rehabilitation was explored to provide evidence for the establishment of disease-specific neurosurgical ERAS protocol for patients with gliomas.

## Materials and methods

### Patient Enrollment

This registry study was conducted in the Department of Neurosurgery, Tangdu Hospital, Air Force Military Medical University (Xi’an, China). The study protocol was approved by the institutional ethics committee of Tangdu Hospital (No. 201907-10) and registered at the Chinese Clinical Trial Registry (ChiCTR1900025108, http://www.chictr.org.cn/showproj.aspx?proj=42016) before implementation. Written informed consent for participation was obtained from all participants. The protocol adheres to the principles outlined in the US Code of Federal Regulations, Title 45, Part 46, Protection of Human Subjects, revised June 23, 2005, and the World Medical Association Declaration of Helsinki.

From September 2019 to May 2021, consecutive patients who underwent elective craniotomy for treatment of glioma at Tangdu Hospital were recruited. The inclusion criteria were as follows: aged 18-65 years old, Karnofsky Performance Score (KPS) ≥70, initially diagnosed supratentorial glioma and lesion involving less than 3 lobes. Patients who needed emergency surgery and with radio- or chemotherapy history were excluded.

From September 2019 to August 2020, patients received a standard neurosurgical care protocol (control group). In June 2020, a neurosurgical ERAS protocol was implemented for glioma patients who received elective craniotomy (ERAS group). Primary outcomes were postoperative hospital length of stay (LOS) and total hospitalization costs. Secondary outcomes were 30-day readmission rate, postoperative complications, duration of the drainage tube, time to first oral fluid intake, time to ambulation and functional recovery status. All patients were followed for 30 days after hospital discharge.

### ERAS Protocol for Gliomas

**Figure 1:**
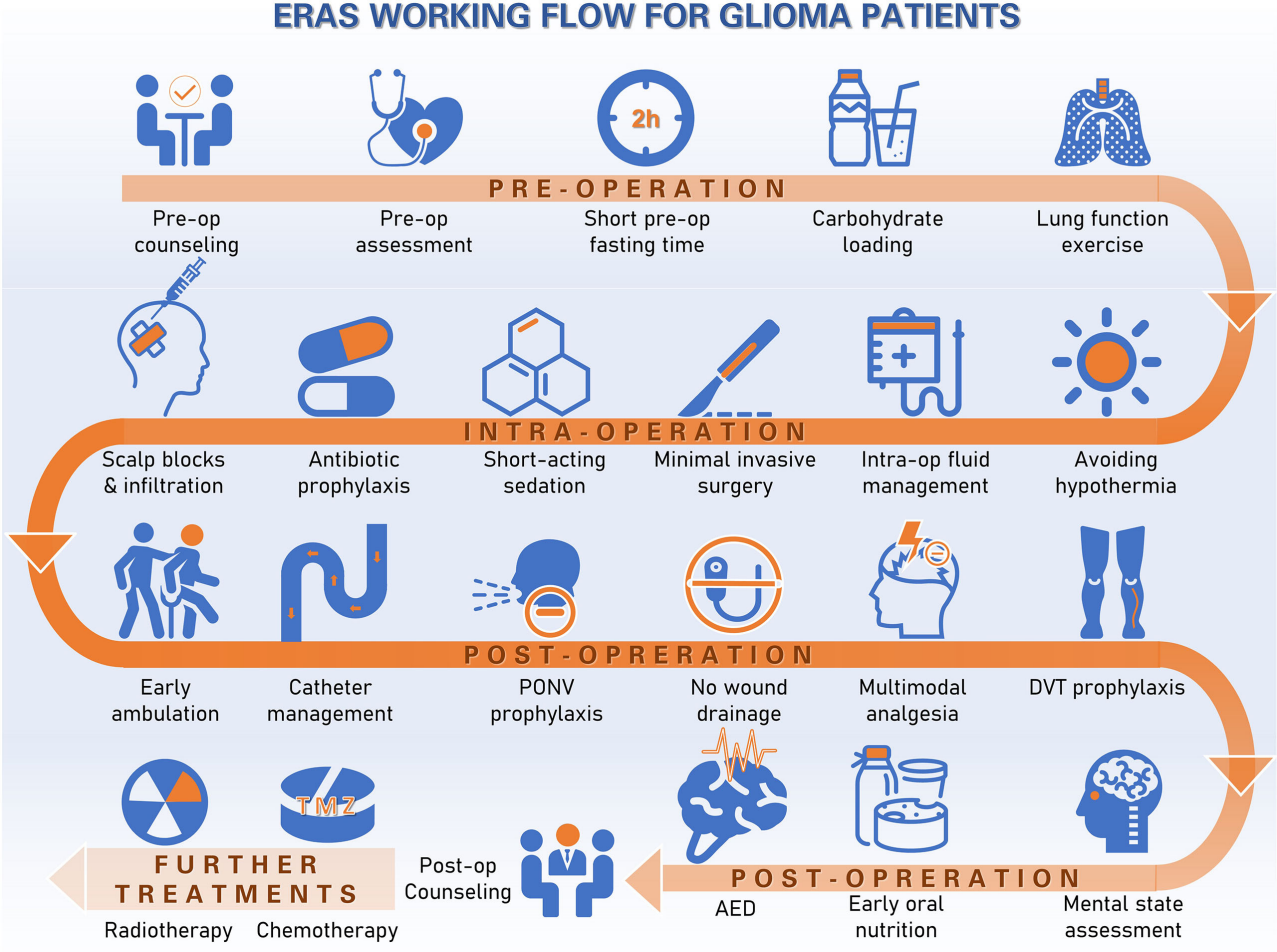
Working flow of ERAS protocol for gliomas.

Details of our institutional ERAS protocol have been previously described (4). The protocol complies with the ERAS society research reporting guidelines (5). Briefly, our ERAS protocol for glioma patients consists of three sections: preoperative, intraoperative and postoperative intervention. Key items include patient and family education, baseline functional and nutritional assessment, smoking and alcohol abstinence, limited fasting, carbohydrate loading, no long-acting sedation, antibiotics prophylaxis, scalp block and local incision analgesia, hypothermia avoidance, goal-directed fluid balance, minimally invasive approaches and techniques for the craniotomy, early termination of the drainage tube, postoperative nausea and vomiting (PONV) assessment, preventive opioid-sparing multimodal analgesia, early mobilization and ambulation, deep vein thrombosis (DVT) prophylaxis and early oral feeding within 24 h. The glioma ERAS protocol was further modified to include extra measures focusing on stressor evaluation, such as mental status. The working flow of ERAS protocol for gliomas was summarized in **Figure 1**.

The hospital anxiety and depression scale (HADS) was applied twice to grade the patient’s psychological status at admission and discharge. HADS has comprised two subcomponents: depression (HADS-D) and anxiety (HADS-A) (6). Changes in HADS scores from admission to discharge were calculated. Minimum clinically important difference (MCID) was also used to detect the changes in HADS scores to assess the mental health of glioma patients. A 1.7-point reduction was set as the indicator value (7).

Perioperative management of patients in the control group was conducted based on the current standard neurosurgical care at our institution. Some elements routinely implemented in clinical practice (e.g., antibiotic prophylaxis, smoking cessation education, etc.) were also applied in the control group.

Patients were discharged according to well-defined discharge criteria, including full consciousness, adequate pain control, body temperature within normal range, ability to take adequate food without the need for intravenous nutrition, effective wound healing and major laboratory tests within normal limits. A discharge readiness assessment was conducted by a senior attending neurosurgeon who was not involved in the study. Clinical or telephone follow-ups were conducted one month after discharge. Adverse events data and KPS scores were collected during follow-up to evaluate the functional status of patients.

The medical record system and the ERAS Record Sheet were used to collect perioperative data. Overall compliance to 20 key ERAS measures was assessed and expressed as a percentage. Good compliance was defined as >80% score per item and/or per patient.

In order to analyze the effect of surgical start time, two surgical cohorts were created based on patients receiving surgery before or after 2 PM. Early cohort (before 2 PM) and later cohort (after 2 PM) were stratified in both groups.

### Statistical Analysis

Continuous variables were expressed as mean ± SD or median [interquartile range (IQR)], and categorical data were presented as numbers (percentages). The student’s *t*-test was used to compare two groups of normally distributed continuous data. The Mann-Whitney *U* test was used to compare differences between non-normally distributed variables. The chi-square tests or Fisher exact tests were used to compare categorical variables. Statistical significance was defined as p<0.05. All statistical analyses were performed using SPSS 22.0 (SPSS Inc., Chicago, IL, USA) and GraphPad Prism 9 (GraphPad Software, San Diego, CA, USA).

## Results

### Patient Sociodemographic and Clinical Characteristics

A total of 166 patients diagnosed with glioma and received neurosurgical treatment were recruited. Nine patients declined informed consent. Five patients were lost clinical follow-up due to long distance or COVID-19 travel restrictions. One patient was lost follow-up due to refuse phone or other connection. A total of 151 patients were included in the final analysis (80 in the ERAS group and 71 in the control group, **Diagram 1**). No significant difference was found in baseline clinical characteristics between the two groups (**Table 1**).

**Table 1:** The demographic characteristics of patients with gliomas in two groups.

Parameters	ERAS (*n*=80)		Control (*n*=71)		*p*
Gender					0.1514	
Male	52	65%	38	53.52%		
Female	28	35%	33	46.48%		
Age					0.1795	
Mean (SD)	51.70	12.21	49.07	11.67		
Median (Range)	56.5	18-65	48	23-65		
Seizure	16	20%	14	19.72%	0.9655	
Partial	12	15%	11	15.49%		
Generalized	3	3.75%	2	2.82%		
Both partial and generalized	1	1.25%	1	1.41%		
Pre-op KPS					0.0641	
100	61	76.25%	55	77.48%		
90	13	16.25%	3	4.23%		
80	3	3.75%	5	7.04%		
70	1	1.25%	2	2.82%		
60	2	2.5%	6	8.45%		
ASA					0.2786	
I, no. (%)	66	82.5%	63	88.73%		
II, no. (%)	14	17.5%	8	11.27%		
Apfel-score					0.1135	
<3	54	67.5%	62	87.32%		
≥3	16	20%	9	12.68%		
Concomitant diseases						
CHD/hypertension	18	22.5%	17	23.94%	0.8338	
Smoker	23	28.75%	28	39.43%	0.1658	
Liver/gallbladder	2	2.5%	3	4.23%	1	
Chronic pulmonary disease	14	17.5%	10	14.08%	0.5667	
Diabetes	11	13.75%	10	14.08%	0.9527	
Miscellaneous	6	7.5%	9	12.68%	0.4302	

^*^SD, standard deviation; ASA, American Society of Anesthesiologists; CHD, coronary heart disease; KPS, Karnofsky Performance Score.

The average age was 51.7 ± 12.21 in the ERAS group and 49.07 ± 11.67 in the control group (p = 0.1795). There were more males than females in both groups, but without notable differences [ERAS group: 52 males (65%) and 28 females (35%); control group: 38 males (53.52%) and 33 females (46.48%)]. No significant difference was found for concomitant diseases (**Table 1**).

For glioma pathology diagnosis, WHO grade IV GBM was the major type in both groups, including 43 cases (53.75%) in the ERAS group and 44 cases (61.97%) in the control group. The tumor location was summarized in **Table 2**. Gliomas mainly invaded the frontal lobe [ERAS group: 19 cases (23.75%); control group: 23 cases (32.39%)], temporal lobe [ERAS group: 12 cases (15%); control group: 7 cases (9.86%)] and parietal lobe [ERAS group: 8 cases (10%); control group: 5 cases (7.04%)]. No significant difference was found between the two groups regarding pathological diagnosis (p = 0.8055).

**Table 2:** Summary of tumor and operation related details.

Parameters	ERAS (*n*=80)		Control (*n*=71)		*p*
Pathology	n	%	n	%	0.1052
Glioblastoma, WHO IV	43	53.75%	44	61.97%	
Diffuse midline glioma, WHO IV	1	1.25%	1	1.41%	
Gliosarcoma, WHO IV	2	2.5%	1	1.41%	
Anaplastic astrocytoma, WHO III	3	3.75%	3	4.23%	
Anaplastic oligodendroglioma, WHO III	1	1.25%	5	7.04%	
Astrocytoma, WHO II	6	7.5%	2	2.82%	
Oligodendroglioma, WHO II	11	13.75%	9	12.68%	
Others, WHO I	21	26.25%	6	8.45%	
Location					0.8055
Frontal	19	23.75%	23	32.39%	
Frontotemporal	6	7.5%	2	2.82%	
Frontoparietal	7	8.75%	7	9.86%	
Fronto-temporal-insula	2	2.5%	3	4.23%	
Fronto-temporal-parietal	0	0%	2	2.82%	
Fronto-corpus callosum	4	5%	5	7.04%	
Temporal	12	15%	7	9.86%	
Temporo-insula	3	3.75%	0	0%	
Temporo-parietal	1	1.25%	1	1.41%	
Temporo-parieto-occipital	1	1.25%	0	0%	
Temporo-occipital	3	3.75%	2	2.82%	
Temporo-thalamus	1	1.25%	0	0%	
Parietal	8	10%	5	7.04%	
Parieto-occipital	2	2.5%	3	4.22%	
Occipital	4	5%	5	7.04%	
Thalamus	3	3.75%	3	4.22%	
Lateral ventricle	4	5%	3	4.23%	
Awake surgery	5	6.25%	1	1.41%	0.2144
Frontal	2	2.5%	1	1.41%	
Frontotemporal	1	1.25%	–	–	
Frontoparietal	1	1.25%	–	–	
Temporo-insula	1	1.25%	–	–	
Surgical length (h)	Median	4.225	Median	4	0.263
	IQR	4-4.225	IQR	3.8-5.0	
Blood loss (ml)	Median	300	Median	200	0.0537
	IQR	200-400	IQR	200-300	
RBC transfusion (ml)	Median	0	Median	0	0.0862
	IQR	0-0	IQR	0-0	

^*^IQR, inter-quartile range.

### Compliance With the ERAS Protocol

**Figure 2:**
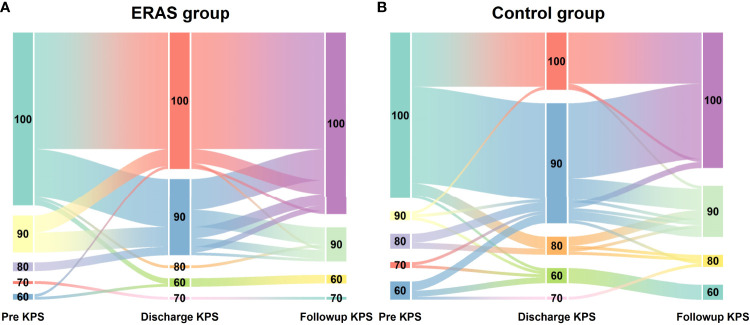
Dynamic changes of KPS scores in glioma patients receiving craniotomy with ERAS protocol **(A)** and conventional care **(B)** during hospitalization.

The target compliance rate for the ERAS protocol in our center was above 80%. Key measures of the ERAS protocol for glioma patients are summarized in **Table 3**. The overall median overall compliance rate was 98% in the ERAS group and 28.17% in the control group (p<0.001). For the purpose of analysis, we also divided the ERAS protocol into pre-operative (5 items), intra-operative (6 items) and post-operative (9 items) measures (**Figure 2**).

**Table 3:** Key measures on ERAS protocol for gliomas and patient compliance.

Measures	ERAS (*n*=80)		Control (*n*=71)		*p*
	n	%	n	%	
1. Preoperative counseling and education	80	100%	48	67.61%	<0.0001
2. Nutritional assessment	75	93.75%	6	8.45%	<0.0001
3. Shortened preoperative fasting time	78	97.50%	21	29.58%	<0.0001
4. Carbohydrate loading	76	95%	0	0%	<0.0001
5. Preoperative pulmonary function exercise	70	87.50%	17	23.94%	<0.0001
6. Scalp blocks and infiltration	80	100%	23	32.39%	<0.0001
7. Antibiotic prophylaxis	80	100%	71	100%	1
8. Short sedation	80	100%	19	22.54%	<0.0001
9. Minimally invasive approaches and techniques for the craniotomy	80	100%	71	100%	1
10. Goal-directed fluid restriction (GDFR) strategy	76	95%	6	8.45%	<0.0001
11. Avoiding hypothermia	79	98.75%	63	88.73%	0.0174
12. PONV prophylaxis	80	100%	19	26.76%	<0.0001
13. No wound drainage	57	71.25%	6	8.45%	<0.0001
14. Preventive opioid-sparing multimodal analgesia	72	90%	32	45.07%	<0.0001
15. DVT prophylaxis	80	100%	66	92.96%	<0.0001
16. Early Off-bed activity and ambulation on POD1	60	75%	17	23.94%	<0.0001
17. Early removal of urinary drainage within 24h	48	60%	0	0%	<0.0001
18. Prophylactic antiepileptic drug therapy	80	100%	71	100%	1
19. Early termination of IV fluid infusion	67	83.75%	7	9.86%	<0.0001
20. Mental state assessment	80	100%	71	100%	1
Overall good compliance items with ERAS protocol*	17	85%	6	30%	0.0011
Median compliance rate	–	98%	–	28.17%	–

PONV, postoperative nausea and vomiting; DVT, deep vein thrombosis; POD, postoperative day; IV, intravenous.

### Primary Outcome Measures

The total hospital LOS and postoperative LOS were significantly shorter in the ERAS group (median: 7 days (IQR: 6-12 days) and 5 days (IQR: 5-7.25 days), respectively) than in the control group (median: 9 days (IQR: 8-14 days) and 7 days (IQR: 7-10 days), respectively) (p<0.0001). However, the median value of total hospitalization cost was 82075.5 CNY (range: 74526-91352 CNY) in the control group and 76538 CNY (range: 69763-89220 CNY) in the ERAS group. However, no significant difference was found between the two groups (**Table 4**).

**Table 4:** Primary outcomes and secondary outcomes between ERAS group and control group.

Parameters	ERAS (*n*=80)		Control (*n*=71)		*p*
Primary outcomes					
Total hospital LOS, days/IQR	7	6-12	9	8-14	0.0003
Postoperative LOS, days/IQR	5	5-7.25	7	7-10	<0.0001
Secondary outcomes					
Total cost of hospitalization, CNY	77003	70023-92121	82076	73969-92042	0.1232
30-day readmission	0	0%	0	0%	–
30-day reoperation rate	0	0%	0	0%	–
Surgical complication, no. (%)					
Mortality	0	0%	0	0%	–
Surgical site infection/subcutaneous effusion	1	1.25%	2	2.82%	0.6011
Intracranial infection	2	2.50%	3	4.23%	0.6663
Epilepsy	1	1.25%	3	4.23%	0.3424
Hemorrhage	0	0%	1	1.41%	0.4702
Intracranial hypertension	1	1.25%	2	2.82%	0.6011
Cerebrospinal fluid leakage	0	0%	1	1.41%	0.3424
Nonsurgical complication, no. (%)					
Respiratory complication	1	1.25%	4	5.63%	0.1876
Cardiovascular complication	0	0%	0	0%	–
Digestive complication	0	0%	0	0%	–
Urinary system complication	1	1.25%	1	1.41%	1
DVT	0	0%	0	0%	–
PONV	6	7.50%	13	18.31%	0.0524

IQR, inter-quartile range; LOS, length of stay; PONV, postoperative nausea and vomiting; DVT, deep vein thrombosis.

### Secondary Outcomes Measures

No mortality or 30-day readmission was found in both groups. Postoperative complications are summarized in **Table 4**. No statistical difference was found in surgical-related complications between the two groups. However, three patients developed an incisional infection or subcutaneous effusion (one in the ERAS group and two in the control group), and five patients (two in the ERAS group and three in the control group) presented with intracranial infection. All these patients recovered following antibiotic treatment wound dressing replacement or lumbar drainage. Other non-surgical-related complications were similar between the two groups. PONV was the most common complication. None of the patients developed DVT.

**Figure 3:**
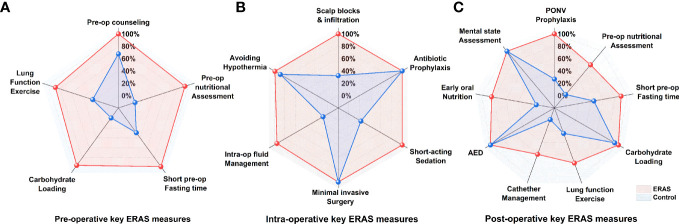
Percent compliance with the ERAS core elements between ERAS groups and control group, categorized by pre-operative **(A)**, intra-operative **(B)** and post-operative **(C)** key measures.

Functional recovery was similar at hospital discharge in both groups (**Table 5**). The median discharge KPS score was 100 (range: 60-100) in the ERAS group and 90 (range: 60-100) in the control group. Although the median KPS score was 100 at 30-day follow-up, no significant difference was observed between groups. The trend of KPS change was also presented in **Figure 3**. Of note, a slow trend of KPS improvement was observed in the control group. The benefits of the ERAS protocol may account for this trend, but further studies with a large sample are needed to verify this hypothesis.

**Table 5:** Key measures on ERAS protocol for gliomas.

Parameters	ERAS (*n*=80)		Control (*n*=71)		*p*
No.	*n*	%/IQR	*n*	%/IQR	
Time to first oral intake, h	6	6-8	10	8-10	0.0018
Time to urinary catheter removal, h					<0.0001
< 24 h	48	60%	0	0%	
24–48 h	30	37.5	22	30.99%	
≥48 h	2	2.5%	49	69.01%	
Wound drainage placement, *n*	23	28.75%	65	91.55%	<0.0001
Time to wound drainage removal, h					
< 24 h	16	69.57%	21	32.31%	
24–48 h	3	13.04%	38	58.46%	
≥48 h	4	17.39%	6	9.23%	
Time to ambulation, days					<0.0001
POD 1	60	75%	17	23.94%	
POD 2	17	21.25%	31	43.66%	
POD 3	2	2.5%	19	26.76%	
> POD 3	1	1.25%	1	1.41%	
Functional status					
Discharge KPS					0.5464
100	48	60.00%	19	26.76%	
90	27	33.75%	40	56.34%	
80	1	1%	6	8.45%	
70	1	1.25%	1	1.41%	
60	3	3.75%	5	7.04%	
30-day follow-up KPS					0.0570
100	64	80.00%	45	63.38%	
90	12	15.00%	17	23.94%	
80	0	0%	4	5.63%	
70	1	1.25%	0	0%	
60	3	3.75%	5	7.04%	

KPS, Karnofsky Performance Score; POD, postoperative day.

Other major ERAS elements were summarized in **Table 5**. For postoperative dietary management, the time to first oral intake was significantly shorter in the ERAS group (6 h, IQR: 6-8 h) compared to the control group (10 h, IQR: 8-10 h; p = 0.0018). A significantly higher percentage of patients in the ERAS group had early urinary catheter removal (within 24 h) (p<0.0001). Postoperative wound drainage tubes were used in a few cases in the ERAS group (23/80, 28.75%) compared to the control group (65/71, 91.55%, p<0.0001). A significantly higher percentage of patients in the ERAS group had early ambulation on postoperative days (POD) 1 and 2 compared with the control group (60/80 vs. 17/71 on POD 1 and 17/80 vs. 31/71 on POD 2, all p<0.0001).

### Mental Health Status

**Figure 4:**
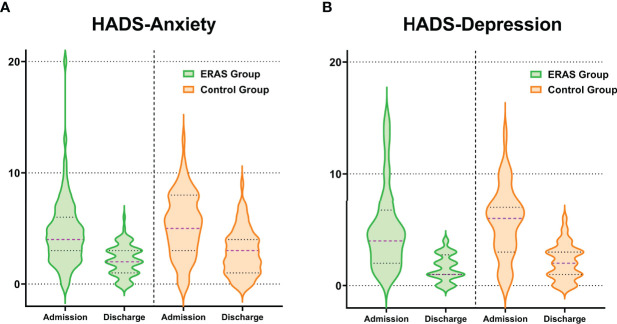
Distribution of changes in HADS-anxiety **(A)** and HADS-depression **(B)** scores at hospital admission and discharge stratified by group allocation.

No significant differences in HADS-A scores were observed between the two groups (ERAS group: -2.54 ± 3.21 points; control group: -2.41 ± 3.20 points, p = 0.8892) (**Table 6**, **Figure 4**). The proportion of patients achieving a clinically relevant improvement (MCID) was also similar in both groups (ERAS group: 69% (55 cases); control group: 59% (47 cases), p = 0.7381).

**Table 6:** Analysis of Hospital Anxiety and Depression Scale (HADS).

Parameters	ERAS	Control	*p*
No. (*n*)	80	71	
HADS-A			
Admission, mean ± SD	4.66 ± 3.06	5.28 ± 2.81	–
Discharge, mean ± SD	2.13 ± 1.33	2.87 ± 2.03	–
Change score, mean ± SD	-2.54 ± 3.21	-2.41 ± 3.20	0.8892
MCID, n (%)	55 (69%)	47 (59%)	0.7381
HADS-D			
Admission, mean ± SD	5.05 ± 3.59	5.72 ± 3.10	–
Discharge, mean ± SD	1.58 ± 1.15	2.11 ± 1.54	–
Change score, mean ± SD	-3.48 ± 3.73	-3.61 ± 3.53	0.5151
MCID, n (%)	59 (83%)	54 (76%)	0.7445

HADS, Hospital Anxiety and Depression Scale; SD, standard deviation; MCID, minimum clinically important difference.

Similarly, no significant differences in HADS-D scores were observed between the two groups (ERAS group: -3.48 ± 3.73 points; control group: -3.61 ± 3.53 points, p = 0.5151) (**Table 6**, **Figure 4**). The proportion of patients achieving a clinically relevant reduction in HADS-D scores was comparable between groups (ERAS group: 83% (59 cases); control group: 76% (54 cases), p = 0.7445).

### Surgical Start Time

**Figure 5:**
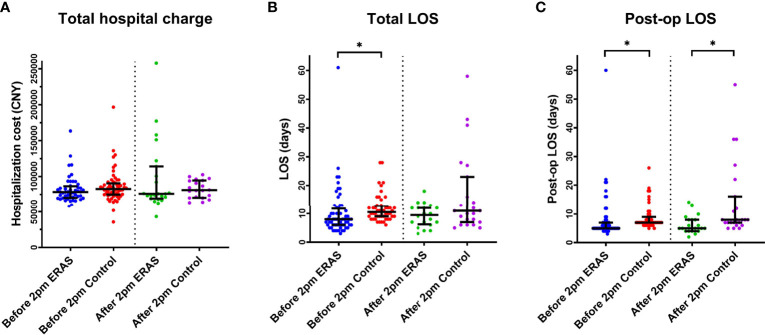
The effect of surgical start time (before or after 2 PM) on both groups on Total Hospital charge **(A)**, Total LOS **(B)** and Post-op LOS **(C)**. *p < 0.05. LOS, Length of stay.

Similar to the article by Sean et al. (8), we set the cut-off point as 2 PM. The demarcation of the 2 PM start time was not arbitrary, but based on the inherent shift changes that impact operating room staff, including nursing, surgical technologists, and anesthetists. In our current study, we did not observe the effect of surgical start time on total hospital cost. However, we noticed that the total LOS in ERAS group was significantly shorter than that in control group (P<0.0005) for the early cohort. There was no difference for the later cohort. As for the post-op LOS, our data demonstrated that the post-op LOS in ERAS group was also shorter than that in control group in both early cohort (p<0.0001) and later cohort (p=0.0048) (**Figure 5** and **Supplementary Tables 1–3**).

## Discussion

The present study has demonstrated a successful implementation of neurosurgical ERAS protocol for glioma patients. To the best of our knowledge, this is the first real-world study describing the neurosurgical ERAS protocol for glioma patients undergoing elective craniotomy. Our results confirmed that this protocol was particularly beneficial for this subgroup of patients, as it shortened postoperative LOS, accelerated rehabilitation recovery and reduced overall complications.

Interestingly, although the ERAS protocol is a quality improvement program, it also influenced the overall survival or progression-free survival, which was mainly determined by the disease features and treatment strategy. Since the initial application of neurosurgical ERAS protocol (4), our ERAS protocol has been continually refined based on the feedback from patients and medical personnel for constant quality improvement. In addition, new series of ERAS protocols for specific diseases subgroups have been developed based on the latest updates from related fields. In the current study, the ERAS protocol for glioma patients was amended to more closely reflect the characteristics of glioma patients, such as mental and neuropsychological changes.

Although the successful application of the ERAS protocol requires a high compliance rate (9, 10), previous studies on the neurosurgical ERAS protocol seldom describe the overall compliance status or partially report results of certain ERAS measures (2, 11). The current registry study tracked the completeness of the ERAS protocol for each patient and obtained 80% compliance for the 20 key measures of the ERAS protocol, including both patient-dependent and provider-dependent measures. Evidence has implied that improved compliance with the ERAS protocol was associated with the improved short-term benefits, such as fewer post-op complications and better functional recovery (12). It is noteworthy that the ERAS protocol was designed as a multidisciplinary clinical procedure, where a single measure cannot achieve cost-effective results without other coordination measures. For instance, early ambulation requires early urinary catheter removal, sufficient postoperative analgesic treatment, PONV prophylaxis, nutritional support and assistance and guidance of the medical staff. Therefore, a high compliance rate in the ERAS group was fundamental for the successful implementation of the ERAS protocol.

As one of the major preoperative management measures, a shorter fasting time with oral carbohydrate intake 2 h before surgery is recommended according to the American Society of Anesthesiologists (ASA) guidelines (13). Our previous study and other similar reports confirmed the benefits of shorter fasting time, including reduced insulin resistance and improved subjective feeling of hunger, thirst and fatigue after surgery (3, 4, 11). In our current study, no cases of aspiration or vomiting were reported during surgery. Similarly, early oral intake to resume gastrointestinal function was encouraged as another key ERAS measure. Together with rapid strategies of postoperative de-escalation of intravenous fluids, early restoration of a normal diet could accelerate perioperative rehabilitation (14, 15). Our current results confirmed this measure to be safe and effective for glioma patients. In addition, early mobilization can reduce the risk of pulmonary complications and DVT, as well as improve cardiopulmonary function (16, 17). In our case series, patients in the ERAS group were encouraged to ambulate on the first day after the operation, which was associated with shorter LOS and better rehabilitation.

Our results also indicated that postoperative LOS and total LOS were significantly reduced in the ERAS group compared to the control group, which is consistent with our previous study and other researches (2, 4). However, no significant differences were observed between the two groups regarding total hospital expenses, although the trend suggested potential economic benefits for the ERAS group. This could be due to the limited sample size. Moreover, no significant difference in postoperative complications (surgical and non-surgical) and KPS scores at discharge between the two groups, underscoring the safety of ERAS protocol.

Previous studies evaluated anxiety and depression in the context of the ERAS protocol in the perioperative period. A *post-hoc* analysis of a previous ERAS study found no effect on preoperative reduction of anxiety and depression measures (18). Another clinical study on total hip arthroplasty did not find any impact of anxiety or depression on functional outcome (19). Since the concept of ERAS involves early mobilization 2-3 h after surgery and early ambulation on POD 1, patients with a strong personalities showed higher pain levels and poorer functional outcomes. Another consideration could be preoperative patient communication and education, which had a positive impact on the patient’s psychological status, reducing the patient’s worries and fears before surgery, thereby resulting in lower admission anxiety and depression scores.

Unlike other previous studies, our results did not support the idea that the later surgical start time (later cohort) itself might associated with longer LOS or higher cost (8, 20). Specifically, our results showed similar total cost, total LOS or post-op LOS inside the same group regardless of surgical start time. However, our results highlighted that the implementation of ERAS might contribute to the reducing the total LOS and post-op LOS, especially for the early surgical start time cases. Considering the mixed factors influencing operation room conditions, such as staff shifting and anesthetic handoffs, we might suggest that the surgical start time might be an important indicator or co-variate to enlarge the effect of ERAS protocol for each case.

A major limitation of the current study is that it was not conducted as a randomized study. However, based on our previous experience, considering the nature of ERAS protocol as a medical care improvement strategy, blinding to study participants and medical care providers could only partially achieved and bias was inevitable. In addition, although our results support the efficacy and safety of the ERAS protocol for glioma patients, due to the differences in medical environment, healthcare provider system and socioeconomic status, multicenter studies with a larger sample size are needed to evaluate its generalizability to different healthcare systems and levels of patient care.

## Conclusion

We have modified and implemented a neurosurgical ERAS protocol for glioma patients. Our results suggest that the application of the ERAS protocol has significant benefits over conventional neurosurgical care. The ERAS protocol accelerated postoperative recovery without additional perioperative risk. However, larger multicenter collaborative research is warranted to evaluate this protocol in relation to the patient’s prognosis.

## Data Availability Statement

The raw data supporting the conclusions of this article will be made available by the authors, without undue reservation.

## Ethics Statement

The studies involving human participants were reviewed and approved by Institutional Ethics Committee of Tangdu Hospital. The patients/participants provided their written informed consent to participate in this study.

## Author Contributions

YuaW and Y-FX wrote the main manuscript text. YuaW, B-FZ, and LW designed the study. NW, P-GJ, S-CG, J-HL, YueW, Y-RX, and Y-FX helped to conduct the study, collected, and analyzed data. NW, S-CG, Y-LZ, and FC provide the service and technical support for this study. G-DG and YQ supervised this study. LW and YuaW prepared tables. All authors reviewed the manuscript. All authors contributed to the article and approved the submitted version.

## Funding

This work was supported by National Natural Science Foundation of China (81601100 and 81772661).

## Conflict of Interest

The authors declare that the research was conducted in the absence of any commercial or financial relationships that could be construed as a potential conflict of interest.

## Publisher’s Note

All claims expressed in this article are solely those of the authors and do not necessarily represent those of their affiliated organizations, or those of the publisher, the editors and the reviewers. Any product that may be evaluated in this article, or claim that may be made by its manufacturer, is not guaranteed or endorsed by the publisher.

## Supplementary Material

Diagram 1 Flow diagram of study design and process.

Supplementary Table 1 Total hospital charge difference based on surgical start time.

Supplementary Table 2 Hospital length of stay (LOS) based on surgical start time.

Supplementary Table 3 Post-operative hospital length of stay (LOS) based on surgical start time.
