# Supplementary figures and images for: Real-World Implementation of Neurosurgical Enhanced Recovery After Surgery Protocol for Gliomas in Patients Undergoing Elective Craniotomy
Source: Front Oncol. 2022 May 24;12:860257. doi: 10.3389/fonc.2022.860257 (PMC9171236; doi:10.3389/fonc.2022.860257)

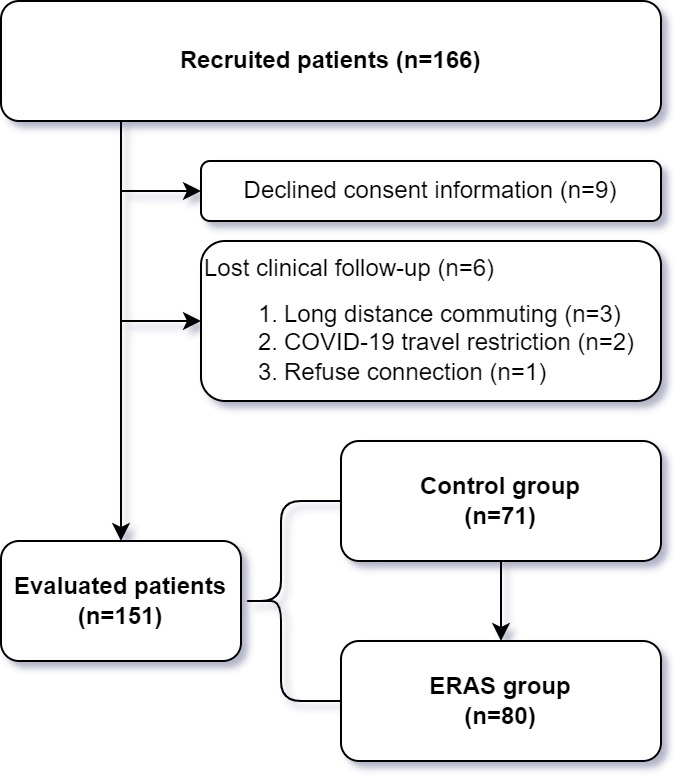

Supplement: Diagram 1 — Flow diagram of study design and process. [file Image_1.jpg]
