# Supplementary material for: Real-World Implementation of Neurosurgical Enhanced Recovery After Surgery Protocol for Gliomas in Patients Undergoing Elective Craniotomy
Source: Front Oncol. 2022 May 24;12:860257. doi: 10.3389/fonc.2022.860257 (PMC9171236; doi:10.3389/fonc.2022.860257)
Supplement: Supplementary Table 1 — Total hospital charge difference based on surgical start time. [file Table_1.pdf]

Sup. Table 1. Total hospital charge difference based on surgical start time

	ERAS Group		Control Group		*p*
	Median	IQR	Median	IQR	
Before 2 PM	78367	70209-86474	82785	74831-90672	0.1058
After 2 PM	75911	69268-114065	81030	70414-94413	0.8566
p	0.9832		0.7534		

IQR: inter-quartile range; CNY: Chinese Yuan.

Sup. Table 2. Hospital length of stay (LOS) based on surgical start time

	ERAS Group (days)		Control Group (days)		*p*
	Median	IQR	Median	IQR	
Before 2 PM	8	6-11.75	10.5	9-12.75	<0.0005
After 2 PM	9.5	6.75-12	11	7.5-19.5	0.1676
p	0.6312		0.9147		-

IQR: inter-quartile range

Sup. Table 3. Post-operative hospital length of stay (LOS) based on surgical start time

	ERAS Group (days)		Control Group (days)		*p*
	Median	IQR	Median	IQR	
Before 2 PM	5	5-7	7	7-9	<0.0001
After 2 PM	5	4-5	8	7-14	0.0048
p	0.4433		0.3299		-

IQR: inter-quartile range
